# Insecticide resistance to permethrin and malathion and associated mechanisms in *Aedes aegypti* mosquitoes from St. Andrew Jamaica

**DOI:** 10.1371/journal.pone.0179673

**Published:** 2017-06-26

**Authors:** Sheena Francis, Karla Saavedra-Rodriguez, Rushika Perera, Mark Paine, William C. Black, Rupika Delgoda

**Affiliations:** 1 Natural Products Institute, Faculty of Science and Technology, University of the West Indies, Mona, Jamaica; 2 Arthropod-borne and Infectious Diseases Laboratory, Department of Microbiology, Immunology and Pathology, Colorado State University, Fort Collins, Colorado, United States of America; 3 Liverpool School of Tropical Medicine, Liverpool, United Kingdom; University of Missouri Columbia, UNITED STATES

## Abstract

The emergence of novel diseases spread by the *Aedes aegypti* mosquito in Jamaica and the Caribbean, has prompted studies on insecticide resistance towards effective management of the vector. Though Jamaica has been using the organophosphate insecticide malathion in its vector control program for more than 30 years, resistance to the pesticide has not been tested in over a decade. We analyzed resistance to malathion and the pyrethroid insecticide, permethrin on mosquitoes collected across St. Andrew, Jamaica, and analyzed the molecular basis of resistance. The Center for Disease Control (CDC) bioassay revealed that *Ae*. *aegypti* mosquitoes from St. Andrew, Jamaica were resistant to permethrin (15 μg/bottle) with mortalities at 0–8% at 30 minute exposure time, while contact with malathion (50 μg/bottle) revealed ≤ 50% mortality at 15 minutes, which increased to 100% at 45 minutes. The standard susceptible New Orleans (NO) strain exhibited 100% mortality within15 minutes. The activities of multifunction oxidases and *p*-nitro phenyl-acetate esterases were significantly greater in most Jamaican populations in comparison to the NO strain, while activities of glutathione-S-transferase, acetylcholinesterase, α-esterase and ß-esterase activity were relatively equal, or lower than that of the control strain. The frequency of knockdown resistance mutations in the voltage dependent sodium channel gene were measured. All collections were fixed for Cys1,534 while 56% of mosquitoes were Ile1,016/Val1,016 heterozygotes, and 33% were Ile1,016 homozygotes. *Aedes aegypti* from St. Andrew Jamaica are resistant to permethrin with variations in the mode of mechanism, and possibly developing resistance to malathion. Continued monitoring of resistance is critically important to manage the spread of the vector in the country.

## Introduction

The incidences of mosquito-borne diseases transmitted by *Aedes aegypti* in the Caribbean have increased. In 2012–2013 the reported cases of dengue virus (DENV), an arbovirus in the family *Flaviviridae*, genus *Flavivirus* was 33,000 [1], followed by the arrival of novel arboviral pathogens, chikungunya virus (CHIKV), family *Togaviridae*, genus *Alphavirus* in 2013 [2] and the Zika virus (ZIKV), family *Flaviviridae*, genus *Flavivirus* in 2015 [3]. Increase in tourism and the ease of travel within the region facilitate the effortless spread and transmission of mosquito-borne diseases throughout the region [2]. Owing to the lack of effective vaccines or medicines to treat most diseases, vector control management using insecticides remains the main form of disease control. However, the vector’s adaptations to insecticides have resulted in insecticide-resistant strains worldwide [4], compromising vector control programs. The mechanisms of resistance include, behavioral changes in biting time, decreased sensitivity to insecticide via lowered insecticide-target site binding interactions through mutations, decreased cuticle penetration of insecticide, and an increase of metabolism of insecticides primarily owing to an elevated increase in expression of enzymes, such as monoxygenases (MFOs), glutathione-S-transferases (GST) and carboxyl-cholinesterase (CCE). MFOs often confer metabolic resistance to pyrethroids, such as permethrin, while GSTs are usually associated with resistance to organochlorides such as DDT. Increased CCE activity usually indicates resistance to pyrethroids, organophosphates and carbamates, such as bendiocarb [4, 5].

The monitoring of insecticide resistance and discerning the underlying mechanisms that contribute to insecticide resistance are crucial in the control of vectors and the spread of their diseases. Some Caribbean countries have undertaken measures to gain an understanding of insecticide-resistance in their mosquito populations as a first step towards monitoring the effectiveness of national vector control programs [6, 7]. Jamaica, however, is yet to engage in a comprehensive examination since early assessments in 1995 [8]. Jamaica has been using the organophosphates, malathion and temephos in its vector control programs since the 1950s to control the transmission of DENV [8]. During a dengue outbreak in 1995, studies to determine resistance to malathion in *Ae*. *aegypti* [9] and the efficacy of ultra-low volume spraying (fogging) to deliver malathion were conducted [8, 10]. The results revealed that this means of delivery was possibly ineffective [10] to populations of mosquitoes determined to be susceptible to malathion as gauged by the WHO filter paper assay [8]. Since then, no plan of action has been implemented to assess the possibility of insecticide resistance, and fogging with malathion, is still being used in vector control [10]. The introduction of CHIKV to the country in 2014 prompted health authorities to switch from malathion to permethrin as a trial adulticide [11]. Given the need for an updated assessment, the current study examined and characterized insecticide resistance in *Ae*. *aegypti* mosquitoes from St. Andrew Jamaica, the most populated parish in Jamaica, with the highest recorded incidence of DENV and CHIKV in the island [12], to the two adulticides currently used in the country. In addition to DENV and CHIKV, St. Andrew also suffered from ZIKV in 2016, with a combined burden from all three diseases increasing four fold from 2010–2016 in suspected cases in St. Andrew and neighboring Kingston [13].

Insecticide resistance in *Ae*. *aegypti* of St. Andrew, Jamaica is reported, via an assessment of metabolic and target site resistance. Activities of key xenobiotic metabolising enzymes, MFO, GSTs and CCEs including alpha-esterases, beta-estereases, *p*-nitrophenyl acetate esterases (*p*NPA) as well as acetyl cholinesterase (AChE) of St. Andrew *Ae*. *aegypyi*, are compared with that of known non-resistant strains. Frequency of mutations Phe1,534Cys and Val1,016Ile in the voltage dependent sodium chanel gene (*vgsc*) associated with pyrethroid resistance in *Ae*. *aegypti* and previously reported in the Caribbean [14, 15] and Latin America [16–18] are also calculated and a status report is provided on resistance in *Aedes* mosquitoes in St. Andrew, Jamaica.

## Materials and methods

### Mosquito collections and rearing

**Fig 1:**
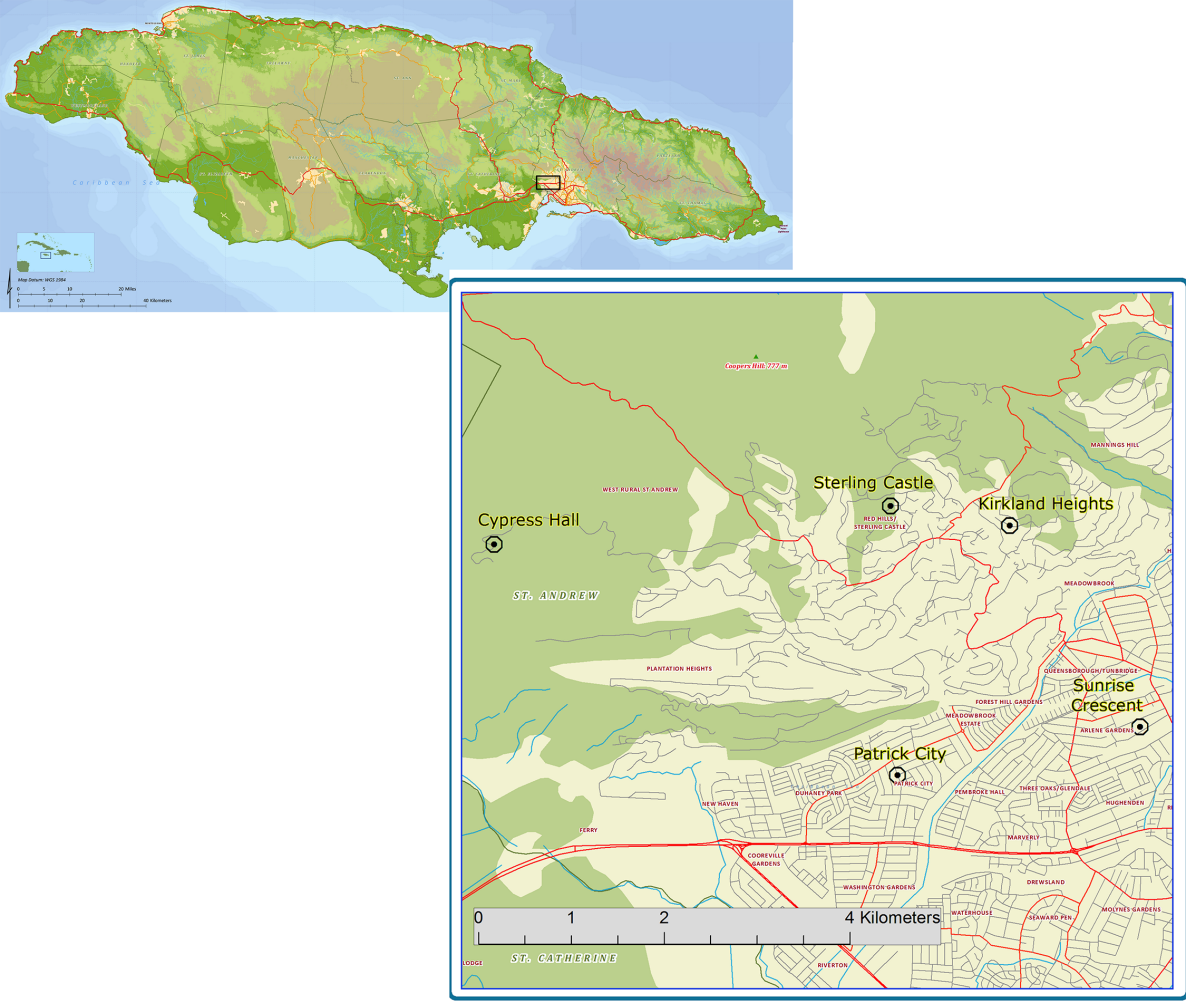
Map of Jamaica, with inset of the areas sampled in the parish of St. Andrew.

Fourth-instar larvae and pupae were collected from a variety of containers found on residential premises (> 20 containers per collection site) from five towns of varying altitudes and economic structures in St Andrew parish, Jamaica in 2015, with permission from the Ministry of Health, Jamaica. The geographic locations of the collection sites are shown in Table 1 and Fig 1. After eclosion, adults were identified to species and *Ae*. *aegypti* females were artificially blood fed (sheep citrated blood) to generate F_1_ and F_2_ offspring. Larvae were fed on liver powder and adults on a 10% sucrose solution and raisins. Insectary conditions were 26–28 ^0^C, 70% humidity and a 14h:10h photoperiod. Two subsamples of 50 unfed mosquitoes per collection site were stored at −80°C for genotype and biochemical activity processing. The remaining F_2_ mosquitoes were used for insecticide bottle bioassays.

**Table 1:** Collection sites with geographical coordinates, altitude in meters and a description of the region.

Sites	Coordinates	Coordinates	Altitude (m)	Rural/City
	(North)	(East)	above sea level	
Sterling Castle Heights	18.060466	-76.836578	300	Rural
Cypress Hall	18.050054	-76.855114	339	Rural
Kirkland Heights	18.057318	-76.827904	332	Rural
Sunrise Crescent	18.037146	-76.817924	57	City
Patrick City	18.030968	-76.838958	18	City

### Bottle bioassays

The susceptibility of Jamaican mosquito populations to the insecticides permethrin and malathion (ChemService, West Chester, PA) were evaluated using the method developed by the Centers for Disease Control and prevention (CDC) [19, 20]. Bottle bioassays were carried out using 3–4 day old females from each collection and the New Orleans susceptible strain (NO) as a reference. Four replicates of 25 unfed adults were exposed to the CDC diagnostic dose of either permethrin (15 μg/bottle) or malathion (50 μg/bottle) and one control bottle pre-coated with acetone [19]. Knockdown was recorded every 15 minutes until 2 hours, however, mortality was reported at 30 minutes. Collections were classified as resistant or susceptible using the updated WHO guidelines [21]: 98–100% mortality indicates susceptibility, 90–97% mortality suggests resistance may be developing, and mortality less than 90% indicates resistance.

### DNA isolation and SNP genotyping

DNA was isolated from individual mosquitoes by the salt extraction method [22], and resuspended in 150 μl of TE buffer (10 mM Tris-HCl, 1 mM EDTA pH 8.0). Single nucleotide polymorphism (SNP) identification for two replacements in the voltage-dependent sodium channel gene (Isoleucine 1,016 and Cysteine 1, 534, from here on referred to as Ile1,016 and Cys1,534 respectively) were genotyped in 50 F_1_ individual mosquitoes, following the allele-specific PCR, melting curve conditions and genotype recording [16, 23]. Genotype frequencies at both loci were tested against Hardy-Weinberg expectations using a χ^2^ goodness-of-fit test with 1 degree of freedom [24]. The Wright's inbreeding coefficient (F_IS_) was calculated using the formula: F_IS_ = 1 - (H_obs_ / H_exp_), where H_obs_ is the number of observed heterozygotes and H_exp_ is the expected number of heterozygote genotypes. An F_IS_ ≥ 0 indicates an excess of homozygotes, whereas an F_IS_ < 0, represents an excess of heterozygotes in the population. The 95% confidence interval was calculated as: *± z*_*0*.*05*_ $\sqrt{\tilde{p}\;(1-\tilde{p})/}n$ [25].

### Biochemical assays and analysis

Enzymatic activity assays were conducted according to Valle, Montella [26]. Briefly; forty females from each population were individually homogenized in 300 μl of deionized water. Homogenate aliquots were transferred to microtiter plates in duplicate. The activities of multiple-function oxidases (MFOs), glutathione-S-transferases (GSTs), α-esterases, β-esterases, p-nitrophenyl acetate esterases (*p*NPA), normal acetylcholinesterase (AChE), inhibited acetylcholinesterase (inAChE) and total protein were quantified colorimetrically. Each enzyme was analyzed in a separate plate. Duplicate values were averaged and all enzyme activity was normalized relative to the total protein in each mosquito. Standard curves for cytochrome-C, α- and β- naphthol, as well as Bovine serum albumin (BSA) were generated to calculate the concentration of cytochrome-C, α- and β-esterases, and total protein respectively in the mosquitoes. All buffer solutions were prepared from analytical grade compounds purchased from Fisher Scientific.

MFO activity was measured via the heme peroxidation method, which uses 3,3’,5,5’ tetramethylbenzidine (TMBZ; Sigma Aldrich, St. Louis, MO) as a substrate. The method indirectly estimates heme-containing enzyme levels in non-blood fed insects [27]. The reaction was conducted in duplicates, in a final volume of 305 μl, which contained 20 μl of the supernatant,17.7 mM potassium phosphate buffer pH 7.2, 0.03% TMBZ/ 123 mM sodium acetate buffer (pH 5), and 0.25% H_2_O_2_. The reactions positive and negative control was cytochrome C (0.01 mg/ml in 250 mM sodium acetate pH 5) and potassium phosphate buffer (90 mM; pH 7.2) respectively. Absorbance was read at 650nm. General oxidase (and heme) content was expressed as micrograms of cytochrome per milligram of protein, by comparing TMBZ oxidation with a standard curve of cytochrome C, prepared using varying known quantities of cytochrome C obtained from bovine heart (Sigma Aldrich, St. Louis, MO).

GST activity was measured using 1- chloro-2, 4-dinitrobenzene (CDNB; Sigma Aldrich, St. Louis, MO) as a substrate. The centrifuged homogenate was incubated in 195 μl of reduced form of glutathione (GSH)/ CDNB mixture (9.5mM GSH in 100 mM potassium phosphate buffer pH 6.5 / 1 mM CDNB in methanol) for 1 hr. The final reaction volume in each well was 210 μl. GST was measured by the change in absorbance as measured at 1 min. intervals for 20 min. at an absorbance of 340nm.

α- or β-esterases were measured indirectly, by their ability to hydrolyze α- or β- naphthyl acetate (Sigma Aldrich, St. Louis, MO) to produce free naphthols, that then forms diazo-dye complexes with Fast blue B (Sigma Aldrich, St. Louis, MO). 200 μl of either α- or β- naphthyl acetate/ sodium phosphate mix (0.3 mM α- or β- naphthyl actetate in 20 mM sodium phosphate buffer pH 7.2) was added to the centrifuged homogenates. The mixture was allowed to react for 15 min., then 50 μl of Fast blue (0.3% Fast blue B in 3.5% SDS; Sigma Aldrich, St. Louis, MO) was added. The mixture further reacted for 5 minutes. The positive control for the reaction was 3.5nmoles/ μl α- or β-naphthol (Sigma Aldrich, St. Louis, MO). Final reaction volume was 260 μl. Absorbance was read at 570nm. 200 μl of 1mM p-nitrophenyl acetate (Sigma Aldrich, St. Louis, MO) in 50mM sodium phosphate buffer pH 7.4, was added to the centrifuged homogenate. The final reaction volume was 210 μl. The reaction was read at 405nm at 15 sec. intervals for 2 minutes.

Acetylcholinesterases were quantified via the Ellman’s method, using dithio-bis-2 -nitrobenzoic acid (DTNB; Sigma Aldrich, St. Louis, MO) and acetylcholine iodine (Sigma Aldrich, St. Louis, MO). Two microtitre plates labeled as ACHE and inACHE were loaded, the final reaction volume was 205 μl, and contained the sample homogenates, 145 μl of a 1% TritonX (Sigma Aldrich, St. Louis, MO) in 100 mM Sodium phosphate buffer pH 7.8, and 10 μl of 10mM DTNB in 100 mM Sodium phosphate buffer pH 7. Acetylcholine Iodine with and without 0.3 mM propoxur (ChemService, West Chester, PA) were added to the plates labeled AChE and inAChE respectively. The mixture was allowed to react for 1 hr and then read at 405 nm. The AChE results are expressed as a percentage of the remaining AChE activity after the addition of propoxur.

Proteins were quantified based on the Bradford method, using a 1:5 dilution of Bio-Rad protein assay dye concentrate (Bio-Rad, Hercules, CA) in water, in comparison to 1 μg/ml BSA (Sigma Aldrich, St. Louis, MO). The mixture was allowed to react for 5 min. and then read at 620nm.

A one way ANOVA was performed in R and pairwise t-tests were used to compare activity means with New Orleans susceptible strain.

## Results

**Fig 2:**
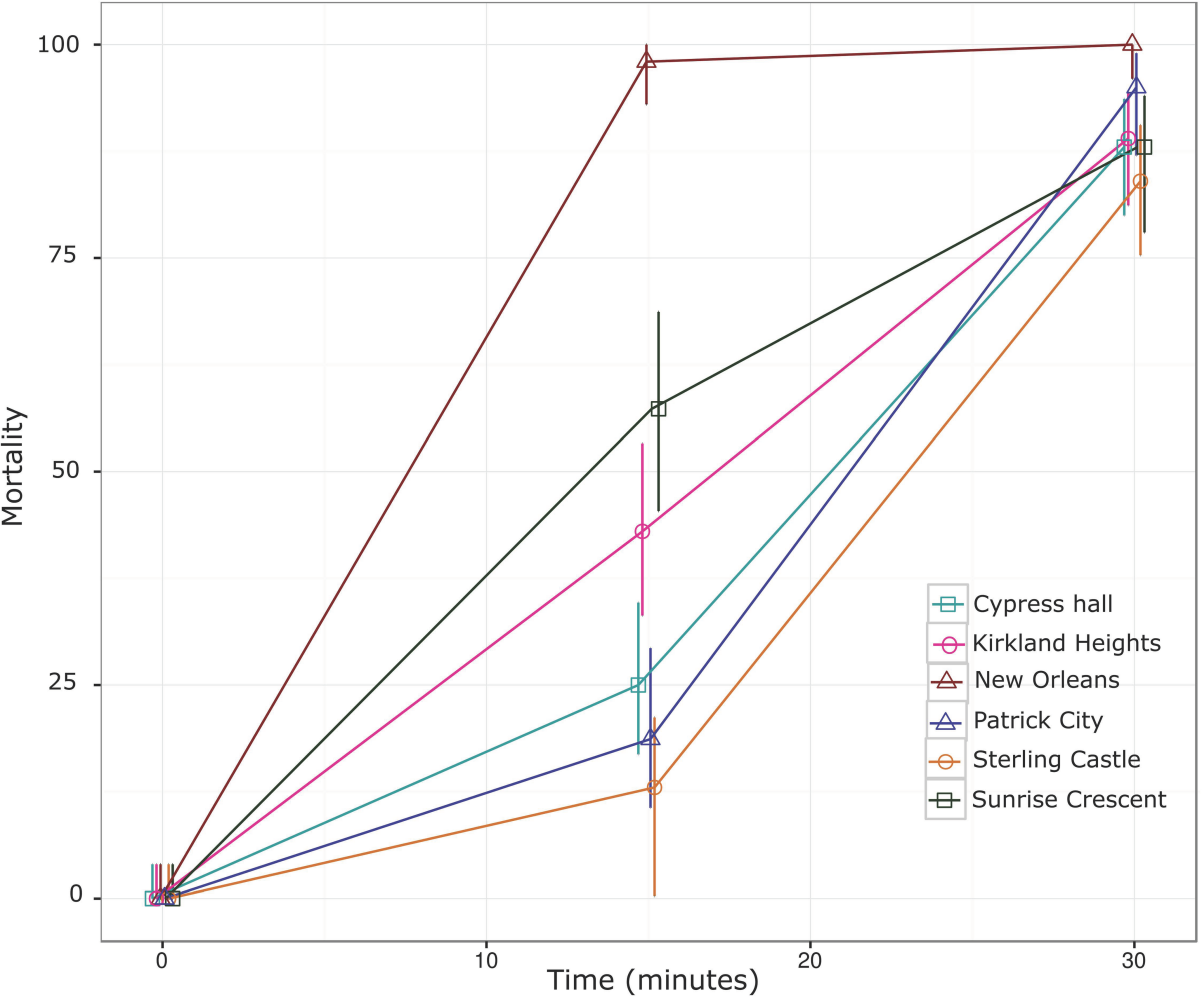
*Aedes aegypti* mortality following exposure to permethrin coated bottles (15 μg active ingredient). Mortality scored at 15 and 30 minutes are shown alongside its 95% confidence intervals. New Orleans (susceptible strain), Cypress Hall, Kirkland Heights, Patrick City, Sterling Castle Heights and Sunrise Crescent.

**Fig 3:**
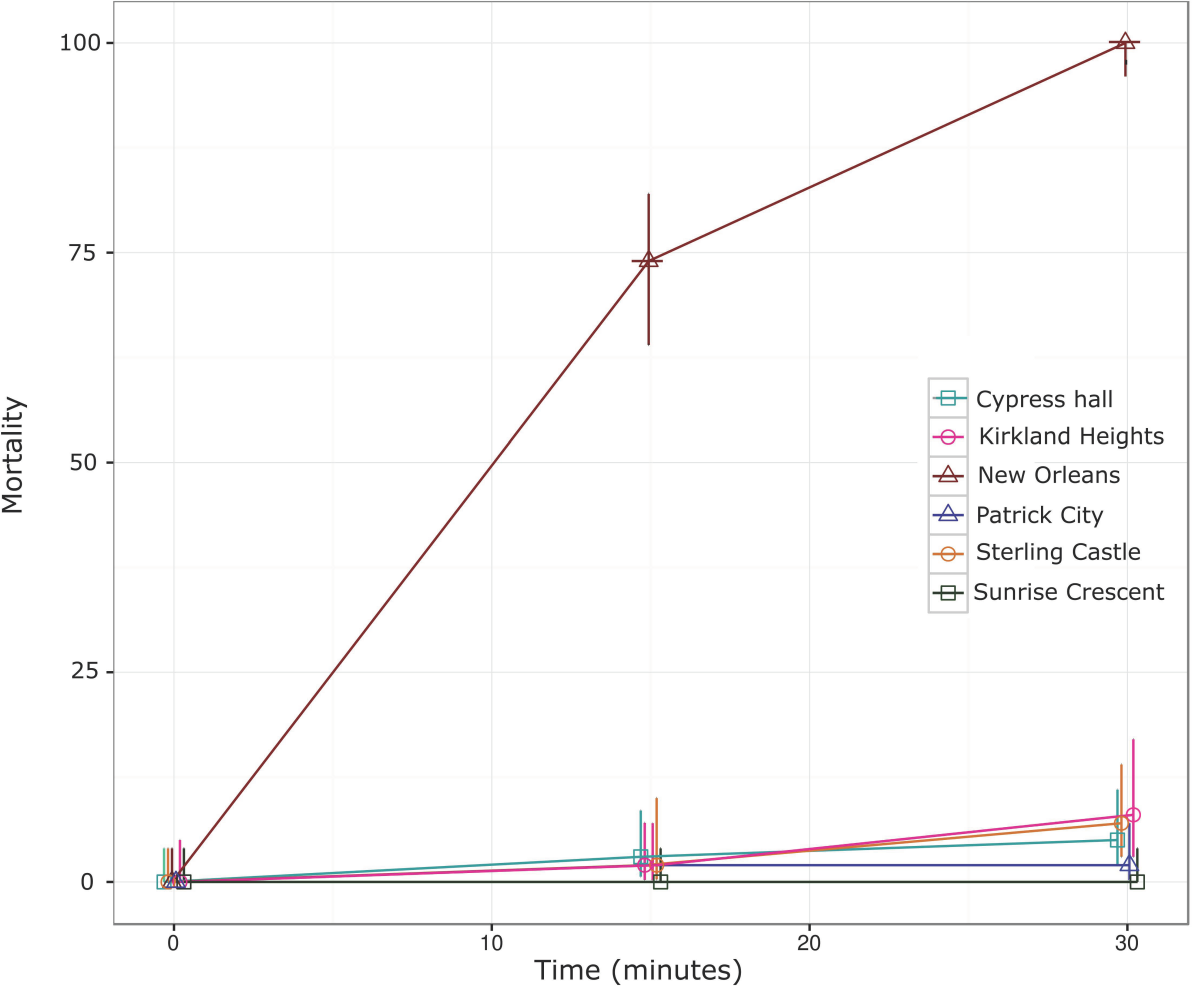
*Aedes aegypti* mortality following exposure to malathion coated bottles (50 μg active ingredient). Mortality scored at 15 and 30 minutes are shown alongside its 95% confidence intervals. New Orleans (susceptible strain), Cypress Hall, Kirkland Heights, Patrick City, Sterling Castle Heights and Sunrise Crescent.

The susceptibility of five mosquito collections to CDC discriminating doses of permethrin and malathion were assessed [19]. The mortality of mosquitoes exposed to permethrin (15 μg) and malathion (50 μg) at 30 minutes is shown in Figs 2 and 3, respectively. All Jamaican mosquito collections were resistant to permethrin, mortalities ranging from 0 to 8%, whereas the susceptible NO strain was completely killed with this dose (Fig 2). In contrast, when exposed to malathion, mortalities ranged from 84 to 90% at 30 minutes (Fig 3). With the exception of Patrick city, the mortality rates at 30 minutes exposure to malathion for mosquitoes collected from St. Andrew, Jamaica were significantly lower (p < 0.05) than the susceptible NO strain. Mortalities increased to 100% for all St. Andrew populations at 45 minutes of exposure.

To confirm and assess the mechanisms of *Ae*. *aegypti* permethrin resistance, the allele frequencies of two common knockdown resistance (*kdr*) mutations in the *vgsc*, were calculated. The Cys1,534 allele was fixed in all populations, whereas the Ile1,016 allele frequencies ranged in frequency from 0.49 in Sunrise Crescent to 0.73 in Kirkland Heights (Table 2).

**Table 2:** Genotype frequencies at residue 1,016 in the voltage gated sodium channel gene in *Aedes aegypti*.

Site Collection		Genotype frequencies			Allele frequency		HW equilibrium	
	N	Ile/Ile	Ile/Val	Val/Val	Ile	χ ^2^	*p*	F_IS_
Sterling Castle Heights	48	23	19	6	0.68	0.43	0.511	0.09
Cypress Hall	48	7	38	3	0.54	16.96	0	-0.59
Kirkland Heights	48	28	14	6	0.73	3.28	0.07	0.26
Sunrise Crescent	48	7	33	8	0.49	6.77	0.009	-0.38
Patrick City	48	14	31	3	0.61	6.33	0.012	-0.36
Overall population	240	79	135	26	0.61			

Ile/Ile homozygotes are resistant, Ile/Val corresponds to heterozygotes and Val/Val are susceptible homozygotes. HW = Hardy Weinberg equilibrium with 1 degree of freedom; and F_IS_ = Wright’s inbreeding coefficient.

**Fig 4:**
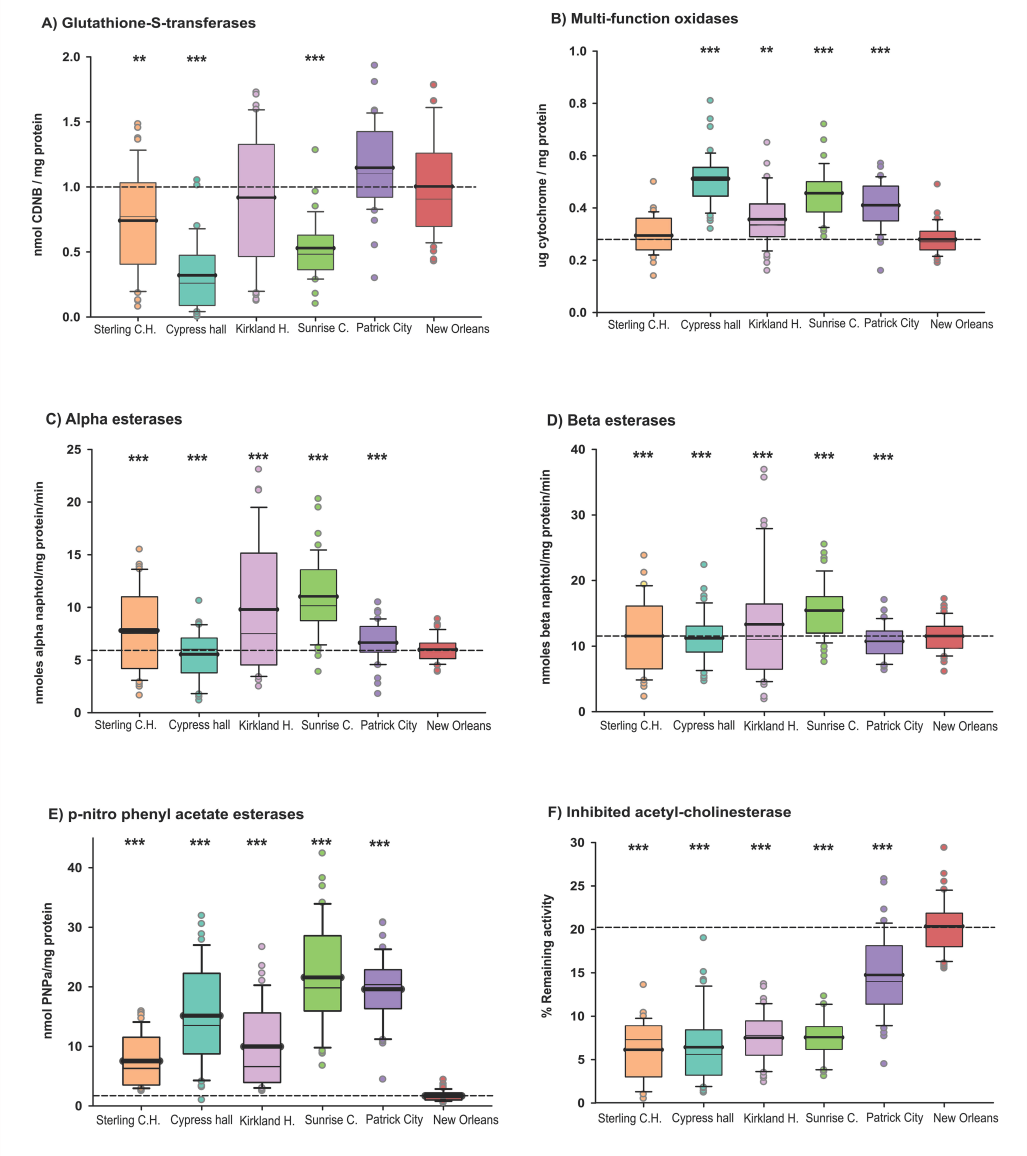
Enzyme activity for *Aedes aegypti* collected from St. Andrew, Jamaica. A) glutathione-S-transferases, B) multi-function oxidases C) alpha esterases, D) beta esterases, E) *p*-nitro phenyl acetate esterases and F) inhibited acetyl cholinesterase obtained for five collection sites in Jamaica (n = 40 per assay). Boxes include the mean (dark line), median (light line), 10^th^, 25^th^, 75^th^ and 90^th^ percentiles. Interrupted line corresponds to the mean activity for the susceptible strain (New Orleans). Asterisks indicate significant difference between the New Orleans and the corresponding strain by a T-test pairwise comparison with pooled SD (*** = p<0.0005, ** = p<0.005 and * = p<0.05).

Metabolic resistance mechanisms were assessed through general biochemical activity assays [26]. Fig 4A and 4B respectively, shows the mean GST activity and MFO quantity in mosquitoes from the five collection sites, pairwise t-tests showed that Sterling City, Cypress Hall and Sunrise Crescent had significantly lower GST activity than New Orleans (Fig 4A). For MFO, Cypress Hall, Kirkland, Sunrise Crescent and Patrick City had significantly more MFOs than New Orleans (Fig 4B). Higher α-esterase (Fig 4C) activity was found in Kirkland and Sunrise Crescent relative to New Orleans, whereas Sunrise Crescent had more β-esterase activity than New Orleans (Fig 4D). The activity of *p*NPA esterases was significantly higher than New Orleans in all Jamaican mosquito collections (Fig 4E). The remaining activity of inAChE was significantly lower in Jamaican mosquito populations than in New Orleans (Fig 4F).

## Discussion

Mosquitoes sampled from five towns in St. Andrew, Jamaica were subjected to either the insecticide permethrin or malathion. The percentage mortality of the mosquitoes was observed every 15 minutes for a period of 2 hrs. The malathion 30-minute mortality score was 84–90% (Fig 3) for all St. Andrew populations, except of Patrick city, which was 95%. Mortality of 100% was observed at 45 minutes. These results may suggest that Jamaican mosquitoes are developing resistance to malathion. According to the CDC bottle bioassay protocol and the WHO recommendations for assessing resistance, mosquito mortality of 80–97% observed using the prescribed dosage and scoring time for a pesticide, suggest possible resistance [19]. Organophosphates such as malathion need to penetrate the cuticles of insects, and be oxidized to a toxic form, malaoxon [28] to be effective at the synapse. The time for cuticle penetration and or oxidation of malathion might vary within mosquito populations, this could explain the longer scoring time to 100% mortality observed in the Jamaican population. Studies conducted between the period of 1995–1996 showed that Jamaican mosquitoes were susceptible or presented low resistance (resistance ratio; RR = 2.2–3.6) to malathion, compared to the control CAREC Trinidad strains (RR = 1) and the most resistant strain Tortola (RR = 14.8) (Rawlins, 1998). The low levels of malathion resistance at the time, were presumed to result from the sporadic use of malathion in the country (Rawlins, 1998). Resistance to malathion has been reported in several studies in Latin America and the Caribbean [8, 29, 30].

In stark contrast, Jamaican collections were highly resistant to the pyrethroid, permethrin (Fig 2). Since pyrethroid used for vector control in Jamaica started only two years ago, the presence of high levels of permethrin resistance is surprising. Other sources of pyrethroid pressure include the use of pyrethroid-based insecticides for house or personal protection, agricultural use [31], or migration of pyrethroid resistant mosquitoes. Jamaican mosquitoes showed high frequencies of two *kdr* amino acid replacements in the *vgsc*. Frequencies of replacement Cys1,534 were fixed (100%) in the population, whereas Ile1,016 was moderate to high (49% - 73%; Table 2). The co-occurrence of both *kdr* mutations has been reported in several Caribbean Islands, including Cayman, Martinique, and Puerto Rico [6, 14, 32]. A recent study, showed that Cys1,534 was required for selection of additional mutations (Val1,016Ile) to confer additional protection to pyrethroids [17]. It seems that these *kdr* frequencies provided complete protection against the CDC permethrin diagnostic dose, since we found no more than 8% mortality in our collections. Cys1,534, which is strongly associated to mechanistic resistance to the organochloride DDT, and pyrethroids, such as permethrin, was detected in the North American region, specifically the Cayman Islands in 2010 [14, 29]. It was also observed in Mexico, with allelic frequencies of 50%, in 2007, where as in 1999, its presence was not detected [33]. The rapid rate at which it is stabilized in the population reflects high fitness in the presence of pyrethroids [17, 33].

The metabolic activity of Jamaican mosquito collections was compared to the NO susceptible reference strain. Altered GST activity was not identified in our samples. Though the organochloride DDT was used in the 1940s up to the 1960s for the eradication of Anopheles *spp*. in Jamaica, in an effort to decrease the spread of malaria in the island; resistance to DDT in *Ae*. *aegypti* larvae collected from Jamaica was observed [34]. GST is an enzyme usually associated with DDT resistance [35]. The use of DDT in vector control in the country is no longer practiced; this may explain the low detection of GST activity in the St. Andrew, Jamaica mosquito population, relative to that of the reference strain (Fig 4A). With the exception of Sterling Castle Heights, the quantity of the oxidative enzymes was significantly greater. MFOs are involved in metabolic resistance to permethrin [36]. The data suggest that the high permethrin resistance observed in mosquitoes collected from St. Andrew, Jamaica may be due to either modification of the *vgsc*, which may prevent pesticide binding with its target site or with elevated oxidative enzymatic activities, which can decrease the amount of pesticide that gets to and binds with their target site. By comparing the frequency of homozygote resistant Ile1,016 allele in the population (Table 2) with the MFO activity levels (Fig 4B) it appears that both resistance alleles are present in mosquito populations across St. Andrew, Jamaica. Mosquitoes of Sterling Castle Heights and Kirkland Heights displayed the highest Ile1,016 frequency, however, their MFO activity was the lowest of the all the five towns sampled across Jamaica. The variation does not seem related to land usage. Of all the carboxyl-cholinesterases analysed (Fig 4C– 4F), *p*NPA esterase activity in all sampled populations appeared the highest in comparison to the laboratory New Orleans strain. Only the Kirkland Heights and Sunrise Crescent populations had higher alpha and beta esterase activity than that of the reference strain. Compared to the NO strain, all Jamaican population sampled (Fig 4F) were sensitive to propoxur at the acetyl-cholinesterase target site, *in-vitro*. The high *p*NPA activity observed in all the St. Andrew population, along with the elevated activity of alpha and beta esterase seen in two of the sampled population could account for the apparent resistance or delay in time to death in the Jamaican population compared to the New Orleans strain. High levels of *p*NPA, alpha and beta esterase activity have been observed in malathion resistant insects [5]. Additionally, the initial inference that different population of mosquitoes may vary in their time to metabolize the insecticide or vary in penetrance time of the insecticide could also be a plausible explanation for the observed apparent malathion resistance.

## Conclusion

Our results show that insecticide resistance to permethrin sprays occur in Jamaica, which were confirmed by both modifications to the voltage sodium gated channel gene and metabolic enzyme activity. The results are very important for the management of *Ae*. *aegypti* population in Jamaica and to control the spread of its diseases. More importantly, the study suggest that various mechanism of resistance are employed by mosquitoes, even over very small geographical locations, and as such, in order for vector control methods to be effective, a non-blanket approach needs to be administered along with constant monitoring of resistance. Surprisingly, malathion may still be a more effective option for *Ae*. *aegypti* control on the island, compared to newly introduced permethrins, despite malathion long use in the country.
