# Correction: Insecticide resistance to permethrin and malathion and associated mechanisms in *Aedes aegypti* mosquitoes from St. Andrew Jamaica

**DOI:** 10.1371/journal.pone.0184387

**Published:** 2017-08-31

**Authors:** Sheena Francis, Karla Saavedra-Rodriguez, Rushika Perera, Mark Paine, William C. Black, Rupika Delgoda

The images for Figs 2 and 3 are incorrectly switched. The image that appears as Fig 2 should be Fig 3, and the image that appears as Fig 3 should be Fig 2. The figure captions appear in the correct order. Please see the correct Figs 2 and 3 here.

**Fig 2:**
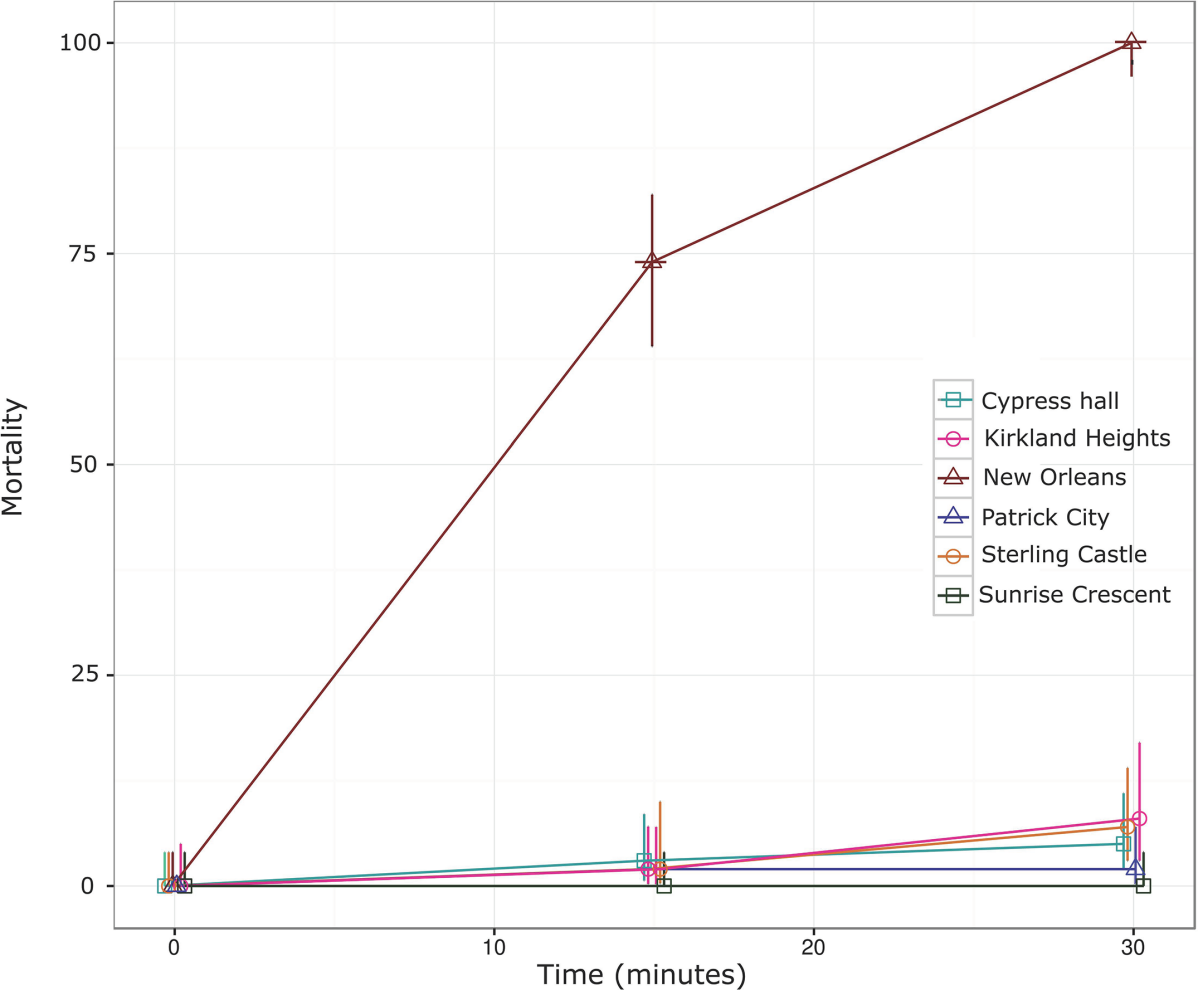
*Aedes aegypti* mortality following exposure to permethrin coated bottles (15 μg active ingredient). Mortality scored at 15 and 30 minutes are shown alongside its 95% confidence intervals. New Orleans (susceptible strain), Cypress Hall, Kirkland Heights, Patrick City, Sterling Castle Heights and Sunrise Crescent.

**Fig 3:**
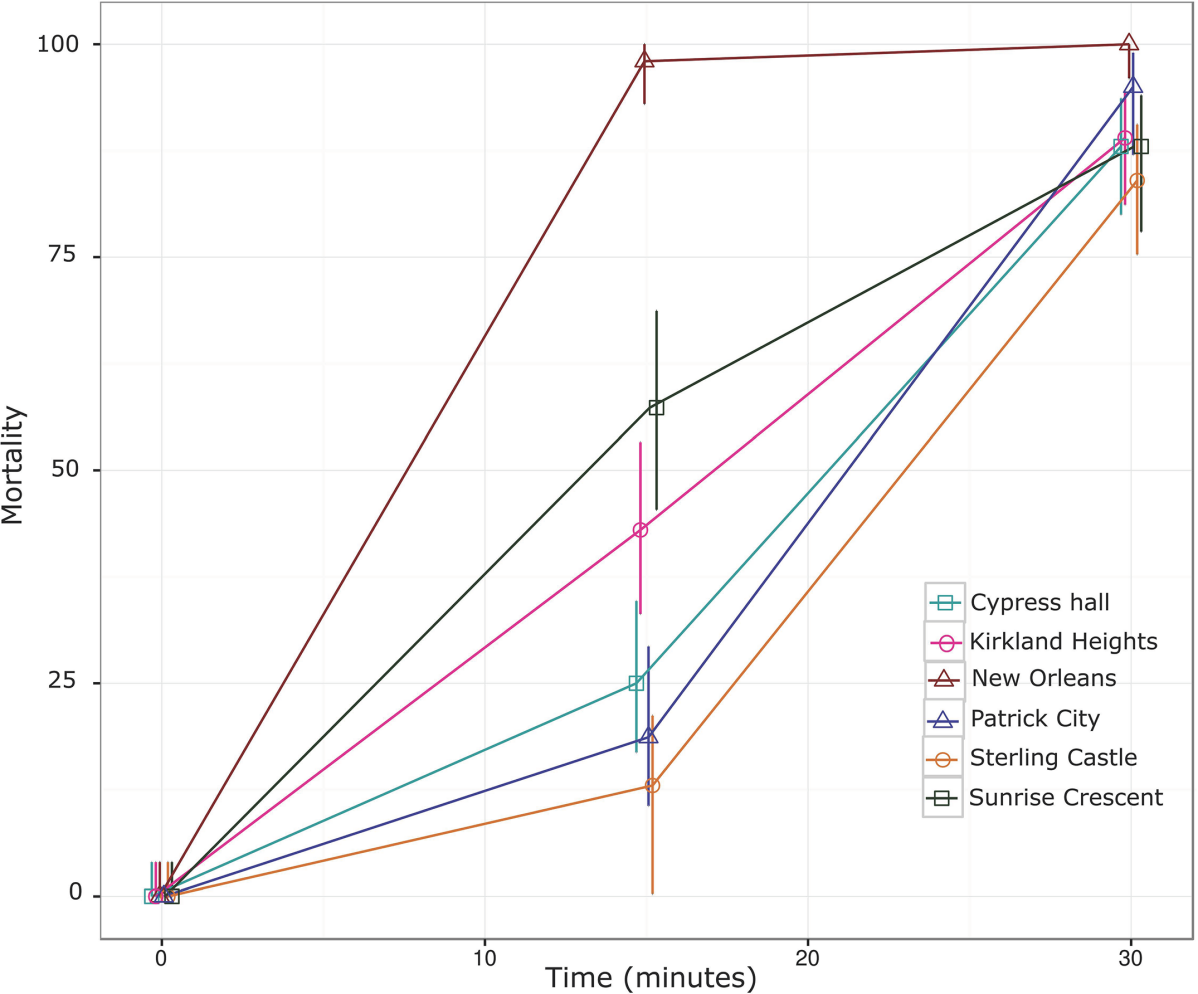
*Aedes aegypti* mortality following exposure to malathion coated bottles (50 μg active ingredient). Mortality scored at 15 and 30 minutes are shown alongside its 95% confidence intervals. New Orleans (susceptible strain), Cypress Hall, Kirkland Heights, Patrick City, Sterling Castle Heights and Sunrise Crescent.
